# Flexible emotion regulatory selection when coping with COVID-19-related threats during quarantine

**DOI:** 10.1038/s41598-021-00716-6

**Published:** 2021-11-02

**Authors:** Maya Shabat, Roni Shafir, Gal Sheppes

**Affiliations:** 1 The School of Psychological Sciences, Tel Aviv University, 6997801 Tel Aviv, Israel; 2 Sagol School of Neuroscience, Tel Aviv University, 6997801 Tel Aviv, Israel

**Keywords:** Human behaviour, Health care

## Abstract

The COVID-19 pandemic poses significant emotional challenges that individuals need to select how to regulate. The present study directly examined how during the pandemic, healthy individuals select between regulatory strategies to cope with varying COVID-19-related threats, and whether an adaptive flexible regulatory selection pattern will emerge in this unique threatening global context. Accordingly, this two-study investigation tested how healthy individuals during a strict state issued quarantine, behaviorally select to regulate COVID-19-related threats varying in their intensity. Study 1 created and validated an ecologically relevant set of low and high intensity sentences covering major COVID-19 facets that include experiencing physical symptoms, infection threats, and social and economic consequences. Study 2 examined the influence of the intensity of these COVID-19-related threats, on behavioral regulatory selection choices between disengagement via attentional distraction and engagement via reappraisal. Confirming a flexible regulatory selection conception, healthy individuals showed strong choice preference for engagement reappraisal when regulating low intensity COVID-19-related threats, but showed strong choice preference for disengagement distraction when regulating high intensity COVID-19-related threats. These findings support the importance of regulatory selection flexibility for psychological resilience during a major global crisis.

## Introduction

If during the last year and a half you witnessed the rapid spread of the COVID-19 pandemic, you probably experienced significant emotional challenges. Your levels of distress may fluctuate from low to high, depending on whether symptom monitoring reveals minor increase in your body temperature versus an emergent major cough, or whether you read less or more discouraging news describing the expected spread of the virus or its influence on the economy. Luckily, however, you can try to ward off the varying levels of distress by flexibly selecting different emotion regulation strategies in different contexts^1–3^. For example, finding out that your fever is 99.3°, can lead you to select reappraisal by telling yourself that low fever is not prototypic of COVID-19 symptom profile, whereas being exposed to horrific news about mounting death rates in Italy, can lead you to select to distract attention by thinking about your home errands.

With mounting numbers of confirmed cases and associated fatalities, the World Health Organization (WHO) declared COVID-19 as a global pandemic^4^. The reality of the crisis led many countries to take drastic measures to fight the mass infection, ranging from social distancing to quarantine and full lockdown. Therefore, beyond the significant health and death tolls, the COVID-19 outbreak has been regarded as a major psychological stressor associated with multiple emotional difficulties. These difficulties range from concrete infection fears, frustration, and anger, to more generalized and severe symptoms of anxiety, depression, and post-traumatic stress^5–8^.

Given the significant emotional toll associated with the COVID-19 outbreak, it is essential to understand how people select to regulate differing affective challenges arising from this pandemic. However, existing empirical evidence remains scarce and indirect, regarding how during the pandemic individuals select between regulatory strategies to cope with differing COVID-19-related threats.

Of existing relevant studies, many did not examine COVID-19-related threats as the *target* of emotion regulation. Specifically, several studies that were conducted during the COVID-19 outbreak, examined the associations between general (non-COVID-19-related) self-reported usage of regulatory strategies and COVID-19 anxiety^9^, post-traumatic stress symptoms^10^, COVID-19 acute stress^11^, quality of life^12^, mental health problems^13^ or COVID-19 risk perception^14^. This shortage of studies examining the regulation of COVID-19-related threats taps on a larger known problem in the field of emotion regulation, involving the use of ecologically limited stimuli (e.g., images of unfamiliar scenes or faces or multiple sounds) that are divorced from individuals’ personally relevant experiences^2,3^ (but see notable exceptions^15,16^).

Importantly, of the very few studies that examined COVID-19-related threats as the target of emotion regulation, no study examined how individuals select between different regulatory strategies to cope with these threats. Specifically, an important longitudinal study that measured the role of self-reported frequency and success of a *single* reappraisal regulatory strategy on subsequent COVID-19 related fear and health behaviors, could not measure the selection between different strategies^17^. Similarly, several recent intervention studies that taught individuals to employ a single regulatory strategy (cognitive reappraisal^18–20^, or self-compassion^20^) also did not examine regulatory selection between strategies.

To fill these gaps, the major aim of the present investigation was to provide unique causal evidence for individuals’ flexible regulatory strategy selection when facing COVID-19-related emotional challenges, during a state issued COVID-19 quarantine. The present study focused on the well-established regulatory selection stage that involves flexibly choosing between available regulatory strategies according to differing situational demands^1,3,21,22^. Within the regulatory selection stage, we concentrated on perhaps the most fundamental finding of the role of differing emotional intensity levels on behavioral choices between regulatory strategies^3^. Specifically, we asked how the experimentally manipulated intensity of COVID-19-related threats influences regulatory selections between strategies that involve disengagement of attention from (i.e., distraction), versus engagement with and re-interpretation of (i.e., reappraisal) emotional information, among healthy individuals during a state issued COVID-19 quarantine.

Given the lack of COVID-19-related stimuli varying in intensity, Study 1 aimed to create a novel set of ecologically relevant sentences covering major, negatively valanced facets of the COVID-19 crisis. These sentences included experiencing COVID-19-related symptoms, threat of being infected, and social and economic consequences of the pandemic. For each facet of the COVID-19 crisis, we created a pair of sentences that vary in threat intensity (low versus high), but that are carefully matched on multiple other dimensions (e.g., topic, word choice, syntactic structure, and sentence length). We expected to validate the novel set of sentences by obtaining increased negative experience ratings for high relative to low intensity sentences, in a sample of healthy Israeli individuals during a state issued COVID-19 quarantine.

Turning to the main goal of the present investigation, in Study 2 we directly examined the influence of emotional intensity levels of COVID-19-related threats on regulatory selection patterns. To that end, we incorporated the stimuli validated in Study 1, in a COVID-19-targeted behavioral Regulatory Selection Task. Specifically, for each of the validated high and low emotional intensity COVID-19-related sentences, participants behaviorally selected whether they prefer to regulate their emotions using disengagement distraction or engagement reappraisal, followed by actively implementing their chosen strategy.

Prior studies^3^ have repeatedly demonstrated an adaptive *flexible* regulatory selection pattern among healthy individuals in a variety of (non-COVID-19-related) unpleasant contexts (e.g., negative images and sounds, electric shocks). Specifically, in high intensity contexts, healthy individuals strongly prefer to disengage attention via distraction, which effectively blocks potent emotional information early, before it gathers force^23,24^. However, in low intensity contexts, healthy individuals strongly prefer to engage with emotional information but to reinterpret its negative meaning via reappraisal, which is both effective in modulating mild emotional reactions, and more beneficial than distraction for long-term adaptation^25,26^.

Our main prediction for Study 2 was to replicate and extend the previously obtained flexible regulatory selection pattern found for non-COVID-19-related stimuli in non-COVID-19-related contexts. Specifically, we expected that healthy individuals during state issued quarantine, will prefer to select reappraisal for low intensity COVID-19-related threats, but prefer to select distraction for high intensity COVID-19-related threats. This prediction was based on two foundations. First, while our investigation was conducted during a stressful quarantine time period, our a-priori sampling decision was to select healthy young individuals, who were at low-risk for developing severe COVID-19 symptoms. As such, these individuals were not expected to demonstrate elevated distress levels (see Table 1 in Supplemental Information for confirmation) which may lead to expect regulatory selection patterns that differ from those obtained in non-COVID-19-related contexts. Second, a central psychological resilience perspective (for review see^27^) and supporting findings from the 2004 tsunami in Southeast Asia, and the SARS pandemic^28,29^, demonstrate that even in the most adverse contexts, the majority of individuals demonstrate adaptive regulatory functioning (see also^30^ for predicted resilience patterns during the COVID-19 pandemic).

## Study 1

### Method

Below we report how we determined our sample size, all data exclusions, all manipulations, and all measures that were collected in both studies. The two studies were approved by the Institutional Review Board of Tel Aviv University and all methods were performed in accordance with the relevant guidelines and regulations. Participants in both studies signed an online informed consent form before starting the experiment.

#### COVID-19 pandemic status

On February 21st 2020, the first case of COVID-19 was confirmed in Israel. During the month that followed, the number of confirmed cases steadily increased, doubling approximately every 3 days^31^. As a result, the state of Israel enforced a strict home quarantine.

Online data was collected on April 21st–22nd, 2020. During these days, all public facilities (e.g., restaurants, airports, schools, public transportation) were shut down, and individuals had to stay at home except for engaging in necessary activities (e.g., getting essential medical or food supplies). When outside, individuals were restricted to a limited distance (i.e., a maximum of 1640 feet, 500 m) from their home and had to wear a face mask. At the time, only essential workers (e.g., hospital and police personnel) were allowed to work outside of home, and the economic activity has decreased sharply by 70%.

#### Participants

Based on a previous study^32^, that also involved validation of two distinct intensities of a new set of emotional stimuli, we set an estimated effect size of η_*p*_^2^ = 0.46 in a formal power analysis, applying the conventional high power of 0.8 and an alpha of 0.05 (notably, the observed effect size η_*p*_^2^ = 0.96 reported below confirmed and exceeded the expected effect size). The formal power analysis pointed to an unacceptably small sample size (i.e., 12 participants). Therefore, we decided to open the online study for two days, during which we aimed to recruit a considerably larger number of participants. By the time two days elapsed, 30 healthy Israeli participants (mean age 26.73 years, 7 men) completed the study online for monetary compensation (15 NIS). None of the participants were excluded from data analysis. 20% of the sample reported that they personally knew someone who had COVID-19. 10% of the sample reported being in one or more risk groups for the COVID-19.

#### Stimuli

We created 15 pairs of short sentences in Hebrew (8–10 words each) that described negatively valanced, self-relevant scenarios related to the COVID-19 crisis. Sentences in each pair varied in their emotional intensity level (high vs. low) but were closely matched on multiple other aspects (e.g., topic, word choice, syntactic structure, sentence length). The scenarios covered major facets of COVID-19-related threats, including experiencing COVID-19-related symptoms (e.g., “Over the last day, your body temperature has risen to *103.5°F*/*99.1°F”* [high/low intensity, respectively]), threat of being infected (e.g., “The delivery person who brought *your*/*your neighbor's* [high/low intensity, respectively] groceries has COVID-19”), and social and economic consequences of the pandemic (e.g., “Due to power load, *significant*/*very minor* [high/low intensity, respectively] disruptions in house electricity service are expected”) (see Supplementary Table 2 for a full list of the sentences). The sentences were written in white font (sized 850X461 pixels) against a black background.

#### Procedure

The study was created using Qualtrics software (Qualtrics, Provo, Utah) and was distributed among designated Facebook paid pool groups. Experimental instructions were administered via pre-recorded video clips (created using Jing software). In these video clips, participants saw short written experimental instructions that were accompanied with detailed auditory explanations. Participants were first explained that they would watch a series of sentences containing negative scenarios related to the COVID-19 crisis, and then rate their level of negative emotional experience in response to each sentence. Participants were taught and practiced (one trial) to allow their emotions and feelings in response to each sentence to arise naturally, without regulating^33,34^. Then, participants performed the Negative Experience Rating Task.

#### Negative experience rating task

The task consisted of 30 trials. Each participant was randomly assigned to one of two predetermined sentence orders. The two sentences' orders were pseudo-randomized, with no more than two consecutive trials of the same emotional intensity, and with at least five trials separating sentences of any given pair.

**Figure 1:**
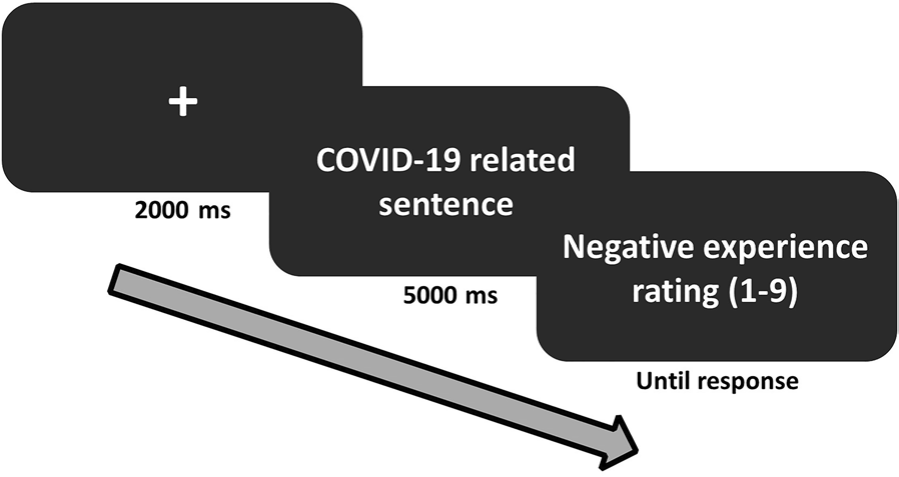
Negative experience rating task. Illustration of a trial structure in Study 1.

Each trial (see Fig. 1) began with a fixation cross (2000 ms), followed by the presentation of a high or low intensity sentence (5000 ms). The offset of each sentence was followed by a nine-point Likert scale (until response) in which participants rated their level of negative emotional experience in response to the sentence (ranging from 1 = "not negative at all" to 9 = "very negative").

To ensure that participants were concentrated in the task, in five trials throughout the experiment, instead of seeing a negative sentence, participants saw a sentence instructing them which number they had to rate in that trial (i.e., attention checks, for example, "in this trail please rate 7"). The average of correct responses approached a perfect score (98.67%, SE = 0.02), with only two participants making a single error.

## Results

**Figure 2:**
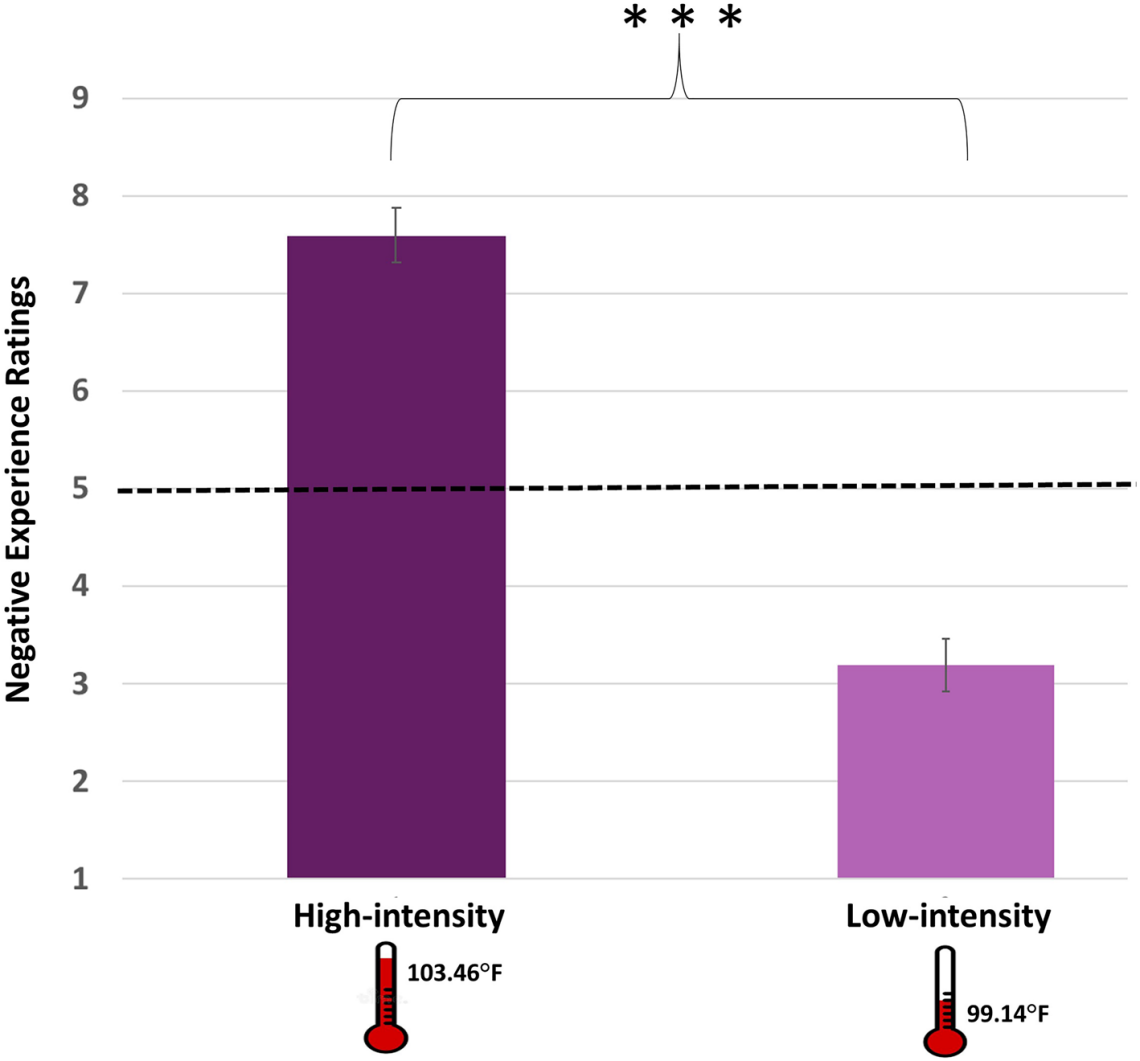
Negative experience ratings for high (dark purple) versus low intensity (light purple) sentences. ***, *p* < 0.001. Bars represent 95% confidence intervals (CIs) for the two conditions. Note that relative to the 5 mid-point of the negative experience scale, negative ratings of high intensity sentences were significantly higher and negative ratings of low intensity sentences were significantly lower.

Consistent with our prediction, high intensity sentences were rated as more negative on average (M = 7.59, SE = 0.15, Cronbach's α = 0.796, 95% Confidence Interval (CI) [7.32, 7.86]), compared to low intensity sentences (M = 3.19, SE = 0.25, Cronbach's α = 0.662, 95% CI [2.91, 3.46]) [*F*(1,29) = 797.53, *p* < 0.001, η_*p*_^2^ = 0.96, see Fig. 2]. Furthermore, we examined whether the average values of negative ratings of the high (M = 7.59) and the low intensity (M = 3.19) sentences, differ from the mid-point of the negative emotional experience scale (i.e., the value 5), that represents intermediate intensity. Indeed, relative to the 5 mid-point of the negative experience scale, negative ratings of high intensity sentences were significantly higher [*t*(29) = 20.50, *p* < 0.001] and negative ratings of low intensity sentences were significantly lower [*t*(29) = − 12.65, *p* < 0.001].

**Table 1:** Means, standard deviations (S.D.s) and comparison between high and low intensity sentences.

Pair number	High intensity Mean (S.D.)	Low intensity Mean (S.D.)	*t*(29)	*p*
1	8.17 (0.73)	3.20 (1.58)	14.88	< 0.001
2	7.03 (1.47)	3.53 (1.93)	7.50	< 0.001
3	7.70 (1.13)	5.20 (2.26)	6.75	< 0.001
4	7.50 (1.67)	4.23 (2.16)	5.58	< 0.001
5	7.83 (1.21)	3.20 (2.06)	11.27	< 0.001
6	7.40 (1.45)	4.80 (2.23)	7.62	< 0.001
7	7.33 (1.64)	2.00 (1.21)	15.53	< 0.001
8	8.73 (0.51)	3.60 (1.76)	15.83	< 0.001
9	8.30 (1.00)	2.13 (1.54)	17.33	< 0.001
10	7.50 (1.57)	2.17 (1.79)	12.82	< 0.001
11	8.13 (1.02)	2.63 (1.99)	14.15	< 0.001
12	7.50 (1.77)	2.90 (2.10)	9.81	< 0.001
13	7.60 (1.43)	2.30 (1.35)	15.93	< 0.001
14	6.50 (1.83)	2.93 (1.73)	9.88	< 0.001
15	6.67 (2.12)	2.97 (1.91)	9.15	< 0.001

Means and SDs of the negative experience ratings across participants for high and low intensity sentences of each of the 15 sentence pairs, as well as *t* and *p* values for the comparison between negative experience ratings of high intensity versus low intensity sentences for each pair.

To provide further evidence for the robustness of our effects, we examined whether differences in negative ratings between high and low intensity sentences are evident for each pair of sentences, across participants. As can be seen in Table 1, in all pairs, the high intensity sentence was significantly higher in negative experience ratings than the corresponding low intensity sentence (all *t*’s ≥ 5.57, *p*’s > 0.001).

For all the reported analyses, we testd whether the assumption of normality is met using the Shapiro–Wilk test. In cases of a normality assumption violation, we re-ran the analysis using a non-parametric test, showing that results remain unchanged (see Supplementary Information for full analyses).

## Study 2

### Method

#### COVID-19 pandemic status

Online data was collected on April 24th–26th, 2020. During these days, the state of Israel had similar strict home quarantine guidelines to those described in Study 1.

#### Participants

Based on the average effect size of the influence of emotional intensity on distraction versus reappraisal preference in previous regulatory selection lab studies^35^, we set an estimated effect size of η_*p*_^2^ = 0.63 in a formal power analysis, applying the conventional high power of 0.8 and an alpha of 0.05 (notably, the observed effect size η_*p*_^2^ = 0.61 reported below confirmed the expected effect size). The formal power analysis pointed to an unacceptably small sample size (i.e., 8 participants). Given that this study was longer than Study 1, in this case we decided to open the online study for three (instead of two) days, during which we aimed to recruit a considerably larger number of participants.

By the time three days elapsed, 92 healthy Israeli participants completed the study online, 70 for monetary compensation (30 NIS), and 22 for course credit. Study 2, that involved learning, practicing and performing a regulatory selection task, was longer and more complex than Study 1. For these reasons, we had additional a-priori exclusion criteria that resulted in exclusion of 17 participants from further analysis (i.e., 18.5% of the sample, see^36^ for similar exclusion rates in a complex online study). Specifically, one participant was excluded due to participation in Study 1. 12 participants were excluded because they had significant completion time delays, defined as 30 min or more than the expected 45 min duration (note that actual average completion duration was 47 min, and that for 11 of these participants, it took 80 min or more to complete the study). Three participants were excluded due to failure to comply with the experimental instructions: when describing how they implemented their chosen strategies (see details below), these participants made more than 50% errors in total and/or more than 50% errors for a specific strategy (i.e., a conservative exclusion criterion in previous emotion regulation studies^33,37^). Last, three participants were excluded because they failed 50% or more of random attention checks (in which they were instructed to press a certain number) that were embedded in a set of questionnaires filled out at the end of the experiment (see details below). Note that repeating all the analyses reported below including these 17 participants left all results unchanged (see Supplementary information for full analyses).

The final sample consisted of 75 participants (Mean age = 23.94 years, 22 men). 22.67% of the sample reported that they personally knew a someone who had COVID-19. 6.67% of the sample reported being in one or more risk groups for the COVID-19.

#### Stimuli

Stimuli were the same 30 high and low intensity ecologically relevant sentences that were validated in study 1.

#### Procedure

The study was created using Qualtrics software (Qualtrics, Provo, Utah) and was distributed among designated Facebook groups. Similar to Study 1, experimental instructions were administered via pre-recorded video clips (created using Jing software), in which participants saw short written experimental instructions accompanied with detailed auditory explanations.

Participants first learned how to implement distraction and reappraisal (two examples for each strategy, order of learning was randomized across participants^33,37^. Distraction instructions involved disengaging attention from the negative content of the sentences by producing unrelated neutral thoughts (e.g., thinking about daily activities, familiar places, or geometric shapes). Reappraisal instructions involved engaging attention with the negative contents of the sentences, but reinterpreting their meaning (e.g., assuming that the situation described in the sentence would improve or thinking about less negative aspects of the situation). During reappraisal, participants were not allowed to think that the negative scenarios described in the sentences were fabricated or unreal^38^, because such reappraisal have been shown to involve disengagement^39,40^. Following the learning phase, participants practiced choosing between the strategies (two trials). After the practice phase, participants performed the Regulatory Selection Task, followed by answering three background questionnaires.

#### Regulatory selection task

The general structure of the Regulatory Selection Task was similar to the standard well-established regulatory selection task^21,35,40^. The task consisted of 30 trials (15 for each emotional intensity). Each participant was randomly assigned to one of two predetermined orders of sentences (different than the two orders used in Study 1). The two sentences orders were pseudo-randomized, with no more than two consecutive trials of the same emotional intensity, and with at least five trials separating sentences of any given pair.

**Figure 3:**
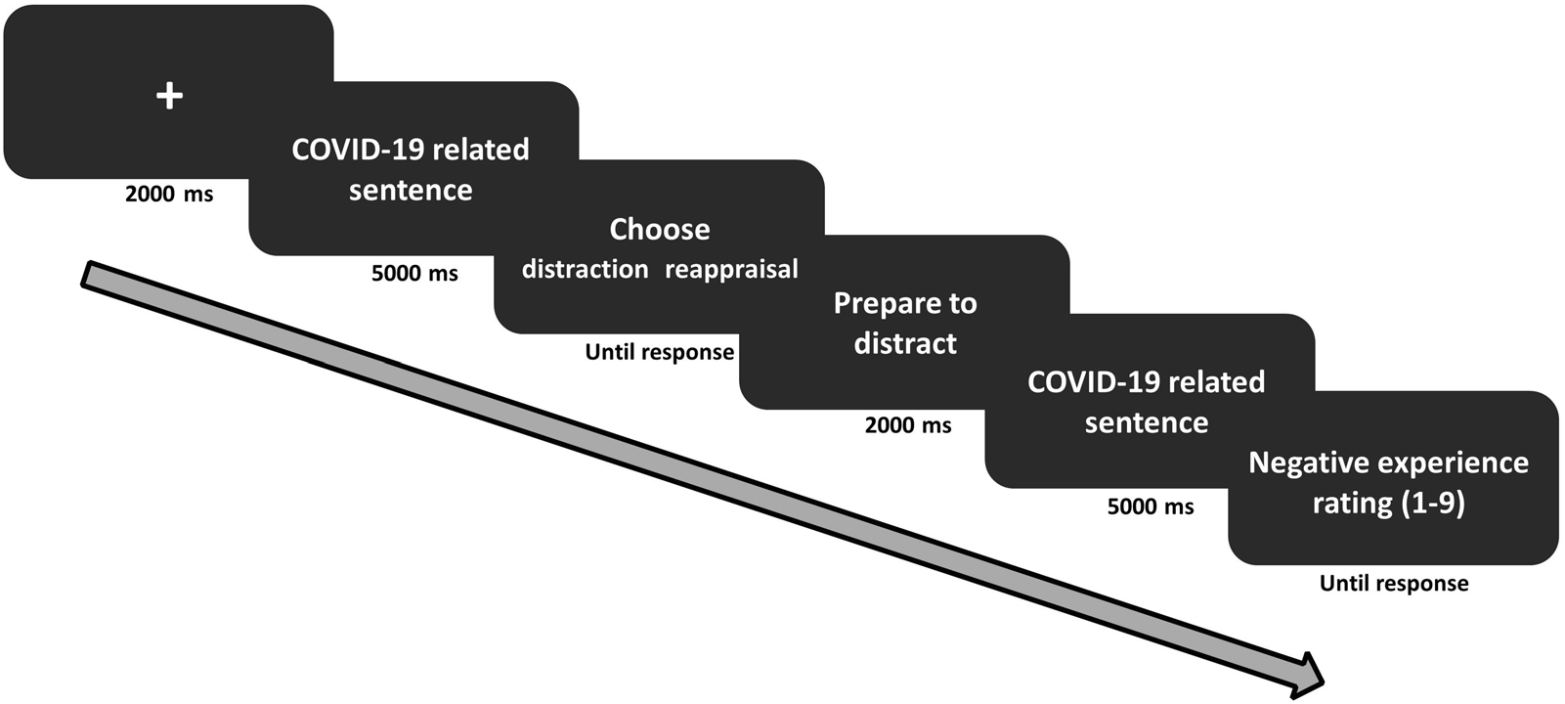
Regulatory selection task. Illustration of a trial structure in Study 2 (an example of a trial where the chosen strategy is distraction).

Each trial (see Fig. 3) began with a fixation cross (2000 ms), followed by the presentation of a high or low intensity sentence (5000 ms). Then, a choice screen was presented (until response), where the two regulatory options, distraction and reappraisal, appeared on different sides of the screen (the side of each strategy was randomly assigned to each participant). Participants were instructed to choose the strategy which they assumed would be more effective in reducing their negative emotional experience in response to the sentence^21,35,40^. Participants indicated their choice by pressing the chosen strategy. Following regulatory choices, the chosen strategy was presented on the screen (2000 ms), followed by the presentation of the same sentence (5000 ms), where participants were instructed to implement their chosen strategy. The offset of each sentence was followed by a nine-point Likert scale (until response) in which participants rated their level of negative emotional experience in response to the sentence (ranging from 1 = "not negative at all" to 9 = "very negative")^2^.

Note that negative emotional experience ratings following regulatory selection and implementation were not analyzed (see Supplementary Information for detailed explanation).

To ensure that participants adhered to regulatory instructions, at the end of eight random trials throughout the experiment (four for each emotional intensity), participants had to write a sentence describing how they implemented the strategy they chose in that trial. A judge who was blind to participants’ chosen strategies coded the sentences as distraction or reappraisal and pointed out to regulatory implementation errors. Levels of successful performance approached a perfect score (97.76%, SE = 0.02).

In order to obtain a general estimate of clinical symptomatology of our sample, at the end of the Regulatory Selection Task, participants completed three questionnaires: the Depression, Anxiety and Stress Scale—21 Items^41^; The Pittsburgh Sleep Quality Index^42^; and a modified version of the six-item Spielberger State-Trait Anxiety Inventory^43^ (see Supplementary Table 1 for all descriptive information, means and standard deviations of the sample).

## Results

**Figure 4:**
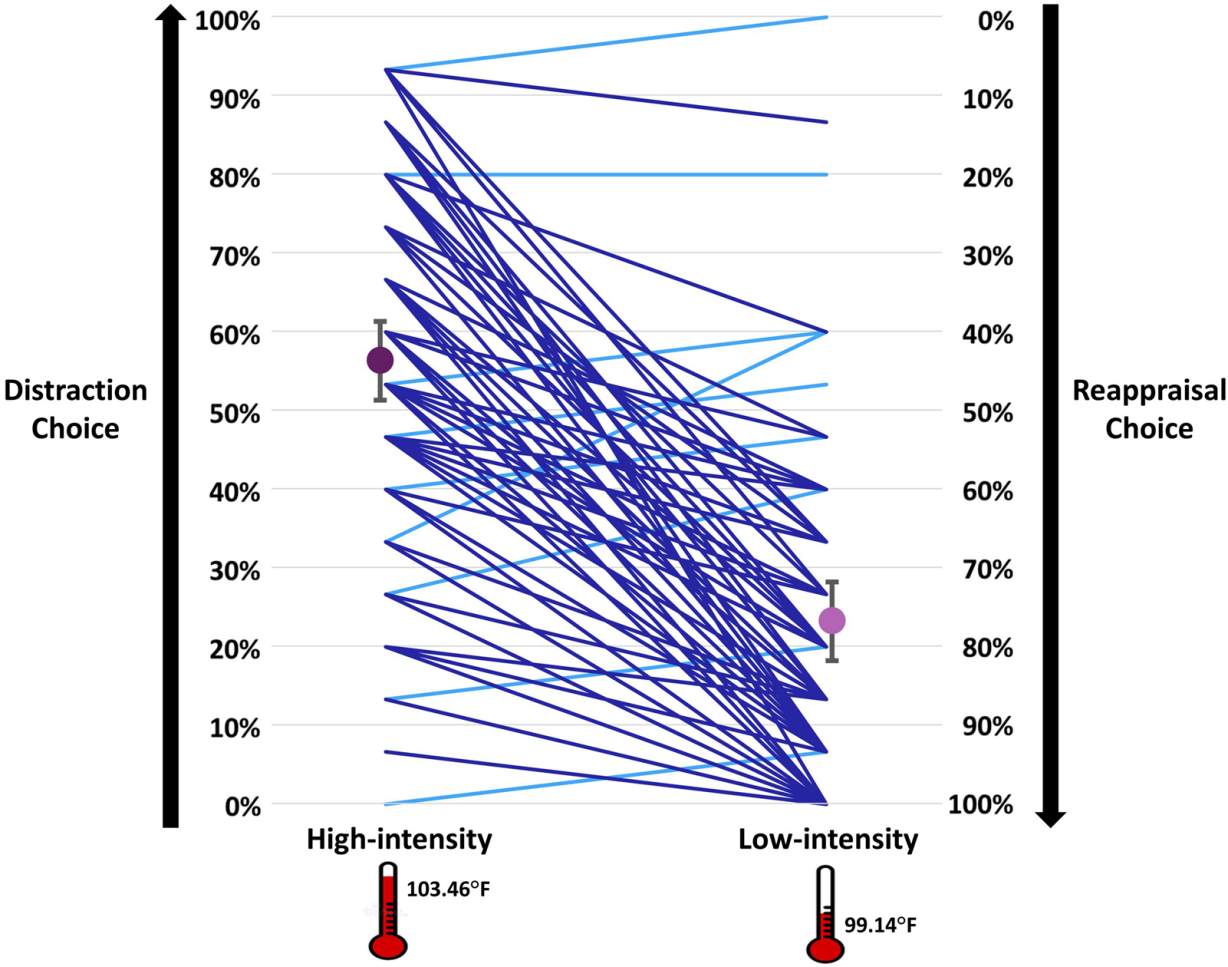
Average regulatory selection preferences for high (dark purple circle) versus low intensity (light purple circle) sentences. Bars represent 95% confidence intervals (CIs) for distraction choice in high intensity (around the dark purple circle) and for reappraisal choice in low intensity (around the light purple circle). For each emotional intensity level, the left y-axis shows distraction choice percentages and the right y-axis shows reappraisal choice percentages (summing up to 100%). The blue lines connect between average distraction choice in high intensity and average reappraisal choice in low intensity for each individual participant. Note that dark blue lines (86.67% of participants) represent the predicted pattern of distraction over reappraisal preference in high intensity, but reappraisal over distraction preference in low intensity, whereas light blue lines represent the opposite pattern.

Providing the first evidence for flexible behavioral regulatory selection patterns when facing COVID-19-related high and low intensity threats, and consistent with prior regulatory selection findings^3^, we found that participants’ preference for distraction over reappraisal increased as emotional intensity increased from low to high intensity [*F*(1,74) = 116.81, *p* < 0.001, η_*p*_^2^ = 0.61] (see Fig. 4). 86.67% (65/75) of the participants showed this flexible pattern of enhanced distraction over reappraisal choice in high relative to low intensity. To provide further evidence for the robustness of our effects, we examined whether differences in regulatory selection between high and low intensity sentences are evident for each pair of sentences, across participants. As can be seen in Table 2, in 93.33% (14/15) of the pairs, distraction over reappraisal preference increased significantly as emotional intensity increased from low to high intensity (all *t*’s ≥ 2.48, *p*’s ≤ 0.015).

**Table 2:** Comparison between percentage of distraction choice in high and low intensity sentences.

Pair number	% distraction choice for high intensity	% distraction choice for low intensity	*t*(74)	*p*
1	61.33	26.67	4.97	< 0.001
2	44.00	24.00	2.55	0.013
3	60.00	50.67	1.31	0.196
4	53.33	12.00	6.85	< 0.001
5	54.67	17.33	5.12	< 0.001
6	41.33	22.67	2.48	0.015
7	54.67	28.00	3.37	0.001
8	70.67	12.00	7.70	< 0.001
9	65.33	17.33	7.50	< 0.001
10	54.67	20.00	4.64	< 0.001
11	68.00	24.00	5.94	< 0.001
12	38.67	16.00	3.24	0.002
13	70.67	21.33	7.40	< 0.001
14	61.33	32.00	4.32	< 0.001
15	45.33	24.00	2.97	0.004

Percentages of distraction choice across participants for high and low intensity sentences of each of the 15 sentence pairs, as well as *t* and *p* values for the comparison between distraction choice percentages for the high intensity versus low intensity sentences for each pair.

Furthermore, we examined whether participants’ preference for distraction in high intensity (M = 56.3%, SE = 2.6%, 95% CI [51.2%, 61.3%]) and for reappraisal in low intensity (M = 76.8%, SE = 2.5%, 95% CI [71.8%, 81.8%]), differ from a 50% no preference rate. Indeed, both the preference for choosing distraction in high intensity [*t*(74) = 2.39, *p* = 0.020] and the preference for choosing reappraisal in low intensity [*t*(74) = 10.93, *p* < 0.001] significantly differed from a 50% no preference rate.

Simmilar to Study 1, for all the reported analyses in Study 2, we testd whether the assumption of normality is met using the Shapiro–Wilk test. In cases of a normality assumption violation, we re-ran the analysis using a non-parametric test, showing that results remain unchanged (see Supplementary Information for full analyses).

## General discussion

The COVID-19 pandemic is associated with significant emotional challenges that need to be regulated. The present two-study investigation provided unique causal evidence for the influence of varying COVID-19-related threats on emotion regulatory selection, among healthy individuals during a state issued COVID-19 quarantine. Study 1 created and validated a novel set of matched high and low intensity emotional stimuli covering major, negatively valanced facets of the COVID-19 crisis. Incorporating these validated stimuli, Study 2 directly examined the influence of emotional intensity levels of COVID-19-related threats on regulatory selection patterns. As predicted, individuals exhibited an adaptive and flexible regulatory selection pattern, manifested in choosing engagement via reappraisal to regulate low intensity COVID-19-related threats, but choosing disengagement via distraction to regulate high intensity COVID-19-related threats.

Given our major aim of testing the influence of emotional intensity on regulatory selection, in Study 1 we created and successfully validated a novel set of COVID-19-related sentences that differ in intensity. All high intensity sentences were rated as more negative, relative to low intensity sentences, in a sample of healthy individuals during a state issued COVID-19 quarantine. These carefully matched sentences provide a relatively pure proxy of the central emotional intensity conceptual construct, that is receiving growing empirical attention across all emotion regulatory temporal stages, including *Identification*^44^, *Selection*^35,45^, *Implementation*^19,46^, and *Monitoring*^37,47^. Moreover, these sentences are ecologically relevant as they tap on concrete emotional challenges individuals were facing during a state issued COVID-19 quarantine. In this regard, they differ from stimuli used in most previous emotion regulation studies, that are divorced from daily experiences (e.g., emotional images) and therefore may not generalize to daily life.

Providing novel evidence, Study 2 found that healthy individuals during a state issued COVID-19 quarantine demonstrate a robust flexible regulatory selection pattern that replicates and extends prior findings^3^. As opposed to most experimental studies in the field of emotion regulation, that entail contexts and stimuli that are removed from individual’s daily lives, the current results demonstrate a robust generalization of a key finding in emotion regulation, in the acute context of a global epidemic, using ecologically relevant COVID-19-related stimuli. Furthermore, most empirical evidence for the importance of emotion regulation during the COVID-19 pandemic^9,10,18^, adopt a traditional theoretical stance that views regulatory strategies as inherently adaptive or maladaptive. Alternatively, our findings, showing context-dependent *flexible* regulatory selection preferences, join a growing consensus of modern theoretical perspectives^1–3^ suggesting that regulatory strategies have different consequences in different contexts.

Obtaining that most individuals demonstrate a flexible regulatory selection pattern involving a strong preference for disengagement from high intensity threats and engagement with low intensity threats, has broad implications. For instance, it may be useful for policymakers who wish to promote people’s engagement with and processing of vital COVID-19 information to refrain from using horrific contents from which individuals may strongly select to disengage.

Emotion regulation flexibility is increasingly perceived as a marker of psychological resilience in the face of adversity^1,30,48,49^. Emotion regulation flexibility can be further broken down to several sub-constructs that correspond to each particular regulatory stage. Specifically, regulatory *implementation* flexibility, namely, the ability to successfully execute different strategies upon demand, has been demonstrated to be important for resilience and long-term adjustment when facing traumatic events^28,50^. Our results add to these prior findings by showing the importance of regulatory *selection* flexibility, namely, the ability to flexibly choose between regulatory strategies, in the face of the current COVID-19 pandemic^30^.

Several limitations of the present study should be noted. First, while we focused on the central regulatory selection stage, it would be crucial to examine other regulatory stages that precede and follow regulatory selection, when facing COVID-19-related threats. For example, future studies could test the importance of the *implementation* stage for regulating COVID-19-related threats, given prior studies showing that flexible implementation ability is associated with healthy adaptation in times of crisis^51,52^.

Second, while the present study was conducted in a country where the COVID-19-related threat was significant and strict quarantine was enforced, it is also worth examining flexible regulatory selection patterns in other countries to look for possible cultural variations. Note, however, that although some differences were observed in regulatory selection patterns between Western and non-Western cultures^50^, other studies^53^ and a recent meta-analysis^54^ demonstrated largely similar regulatory selection patterns among different cultures.

Third, our sample consisted of healthy young individuals, who were generally at low-risk for developing severe COVID-19 symptoms and had relatively low levels of depression, anxiety, stress, and sleep symptoms. Future studies should examine regulatory selection among vulnerable high-risk and clinical populations.

Fourth, the validation of our novel set of ecologically relevant COVID-19-related sentences in Study 1 was obtained via self-reports of emotional intensity, which may be prone to reporting biases. While possible, the self-reported intensity categorization in Study 1 was highly predictive of *behavioral* regulatory selection patterns in Study 2. This finding suggests that reporting biases are unlikely to be the sole driver of our intensity categorization. However, future studies should cross-validate the intensity categorization of our COVID-19-related sentences using alternative performance-based measures (e.g., using peripheral physiology, electrophysiology).

Last, the present investigation used two of the most commonly used engagement and disengagement regulatory strategies, namely distraction and reappraisal^3^. While important, future studies should examine the regulatory selection of other strategies in dealing with personally relevant COVID-19-related threats.

## Supplementary Information

Supplementary Information 1.

Supplementary Information 2.

Supplementary Information 3.

Supplementary Information 4.

## Data Availability

All data files analyzed for both studies are included in the Supplementary Information.
