# Supplementary material for: Flexible emotion regulatory selection when coping with COVID-19-related threats during quarantine
Source: Sci Rep. 2021 Nov 2;11:21468. doi: 10.1038/s41598-021-00716-6 (PMC8563799; doi:10.1038/s41598-021-00716-6)
Supplement: Supplementary file 1 — Supplementary Information 1. [file 41598_2021_716_MOESM1_ESM.pdf]

Supplementary Information

Flexible Emotion Regulatory Selection When Coping with COVID-19-Related Threats During Quarantine

Maya Shabat, Roni Shafir and Gal Sheppes

Study 2: Complementary analyses including all excluded participants (n=92)

Providing the first evidence for flexible behavioral regulatory selection patterns when facing COVID-19-related high and low intensity threats, and consistent with prior regulatory selection findings[1], we found that participants' preference for distraction over reappraisal increased as emotional intensity increased from low to high intensity [$F(1,91) = 151.01$, $p < .001$, $\eta_p^2 = 0.62$]. 85.87% (79/92) of the participants showed this flexible pattern of enhanced distraction over reappraisal choice in high relative to low intensity. To provide further evidence for the robustness of our effects, we examined whether differences in regulatory selection between high and low intensity sentences are evident for each pair of sentences, across participants. In 93.33% (14/15) of the pairs, distraction over reappraisal preference increased significantly as emotional intensity increased from low to high intensity (all t's ≥ 2.68, p's $\leq .009$).

Furthermore, we examined whether participants' preference for distraction in high intensity (M = 58.6%, SE = 2.4%) and for reappraisal in low intensity (M = 75.5%, SE = 2.4%), differ from a 50% no preference rate. Indeed, both the preference for choosing distraction in high intensity [$t(91) = 3.65$, $p < .001$] and the preference for choosing reappraisal in low intensity [$t(91) =10.66$, $p < .001$] significantly differed from a 50% no preference rate.

These findings show that all of the reported analyses in the main manuscript were uninfluenced by the exclusion of participants.

Complementary analyses using non-parametric tests

The Wilcoxon signed-rank test is appropriate when there is a non-normal distribution and the data is paired[2]. Below we report the relevant analyses using Wilcoxon signed-rank test, showing that results are left unchanged.

Study 1:

Consistent with our predictions, high intensity sentences were rated as more negative, compared to low intensity sentences (W = 465.00, $p < .001$). We also tested whether differences in negative ratings between high and low intensity sentences are evident for each pair of sentences, across participants. In all pairs, the high intensity sentence was significantly higher in negative experience ratings than the corresponding low intensity sentence (all W's ≥ 325.00, *p*'s < .001).

Study 2:

Consistent with our predictions, we found that participants' preference for distraction over reappraisal increased as emotional intensity increased from low to high intensity (W = 3885.00, $p < .001$). To provide further evidence for the robustness of our effects, we examined whether differences in regulatory selection between high and low intensity sentences are evident for each pair of sentences, across participants. In 93.33% (14/15) of the pairs, distraction over reappraisal preference increased significantly as emotional intensity increased from low to high intensity (all W's ≥ 570.00, *p*'s ≤ .01). Furthermore, we examined whether participants' preference for distraction in high intensity (Med = 60%) and for reappraisal in low intensity (Med = 80%) differ from a 50% no preference rate. Indeed, both the preference for choosing distraction in high intensity (W = 2,977.50, $p = .001$) and the preference for choosing reappraisal in low intensity (W = 365.00, $p < .001$) significantly differed from a 50% no preference rate.

Study 2: Explanation of why negative emotional experience ratings following regulatory selection and implementation were not analyzed

Negative emotional experience ratings following regulatory selection and implementation were not analyzed because they are un-interpretable in Regulatory Selection Tasks[1]. First, since participants freely choose their preferred strategy, and tend to strongly prefer distraction versus reappraisal in high versus low intensity, the number of trials in which participants eventually implemented each strategy in each emotional intensity is uneven. Second, and more importantly, inferences about differential effectiveness of distraction versus reappraisal require equating stimuli's intensity level for each of these conditions. This is not possible in Regulatory Selection Tasks because participants tend to choose distraction over reappraisal as emotional intensity increases[1]. Therefore, distracted sentences are presumably more intense than reappraised sentences from the outset and are thus incomparable.

Study 2: Background questionnaire data of the sample

Table 1. *Averaged Sums and Standard Deviations (SDs) for Questionnaires*

questionnaire	Averaged Sum	SD
DASS-21		
Total Score	12.91	10.26
Depression	4.52	4.10
Anxiety	2.20	3.04
Stress	6.19	4.36
PSQI (sleep quality)	5.44	3.12
STAI-s (adapted for COVID-19 see below)	15.07	4.07

Table 1. The table displays means and standard deviations for the questionnaires that were administered at the end of Study 2. The DASS-21 is a set of three self-report sub-scales (each scale contains 7 items), designed to measure the emotional states of depression, anxiety and stress[3]. The Depression, Anxiety, Stress and total DASS values presented in the table represent the averaged sum of each sub-scale, across participants. The PSQI is a self-report questionnaire assessing sleep quality over a 1-month time period[4]. The PSQI values presented in the table represent the averaged sum of the seven sleep quality component scores, across participants. The STAI-s is a 6-item self-report scale designed to measure feelings of worry, tension, apprehension, and nervousness individuals are currently experiencing (i.e., state anxiety)[5]. For Study 2, we adapted this scale for our purposes by adding the suffix "during the COVID-19 period" to each item (e.g., “I feel at ease *during the COVID-19 period*”). The STAI values presented in the table represent the averaged sum of these 6 items, across participants.

Table 2. *Pairs of high and low intensity sentences presented in Studies 1 & 2*

	Low intensity	High intensity
**1**	Over the last day, your body temperature has risen to *99.1°F*.	Over the last day, your body temperature has risen to *103.5°F*.
**2**	A man in a seat *far away from* you on the bus had a *mild* cough.	A man in the seat *next to* you on the bus had a *persistent* cough.
**3**	During the last few hours, you have experienced *minor* difficulty breathing.	During the last few hours, you have experienced *severe* difficulty breathing.
**4**	Your primary physician who treated you two *months* ago has COVID-19.	Your primary physician who treated you two *days* ago has COVID-19.
**5**	A COVID-19 patient sat at the same restaurant table as you did a *month* before your visit.	A COVID-19 patient sat at the same restaurant table as you did an *hour* before your visit.
**6**	The delivery person who brought *your neighbor's* groceries has COVID-19.	The delivery person who brought *your* groceries has COVID-19.
**7**	Your blood type was found to be *minimally* associated with severe COVID-19 symptoms.	Your blood type was found to be *highly* associated with severe COVID-19 symptoms.
**8**	Your uncle is staying in a *quarantine site** due to a *minor* COVID-19 health condition.	Your uncle is *hospitalized* due to a *critical* COVID-19 health condition.
**9**	Experts estimate that a mutation of the COVID-19 virus will be *non-lethal*.	Experts estimate that a mutation of the COVID-19 virus will be *lethal*.
**10**	Experts estimate that the transmission rate will *decrease significantly* in the coming *month*.	Experts estimate that the transmission rate will *not decrease* in the coming *year*.
**11**	Hospitals predict that the healthcare system will *be minimally affected* due to the load.	Hospitals predict that the healthcare system will *collapse* due to the load.
**12**	You have been updated that you need to undergo a COVID-19 test as *part of a random sample.*	You have been updated that you need to undergo a COVID-19 test out of a *valid concern that you have been infected.*
**13**	Last night you woke up *once* because of a very *mild* cough.	Last night you woke up *many times* due to a very *serious* cough.
**14**	Due to power load, *very minor* disruptions in house electricity service are expected.	Due to power load, *significant* disruptions in house electricity service are expected.
**15**	An announcement has been posted, alerting that a *minor* internet service slowdown is expected.	An announcement has been posted, alerting that a *crash* in internet service is expected.

*During the COVID-19 crisis in Israel, people who had COVID-19 but did not show severe symptoms or a need for hospitalization could choose to spend their isolation time in designated "quarantine sites" like special hotels.

**Note that translating from Hebrew (the language the experiment was administed) to English requires certain necessary changes in length and wording in order to preserve the original meaning.

References

1. Sheppes, G. Transcending the “good & bad” and “here & now” in emotion regulation: costs and benefits of strategies across regulatory stages. *Advances in Experimental Social Psychology*, **61,** 185-236 (2020).
2. Graham, A., Hampton, M. & Willett, C. What not to write: An intervention in written communication skills for accounting students. *Int. J. Manag. Educ.* **8,** 67–74 (2010).
3. Lovibond, P. F., & Lovibond, S. H. The structure of negative emotional states: Comparison of the Depression Anxiety Stress Scales (DASS) with the Beck Depression and Anxiety Inventories. *Behav. Res. Ther.* **33,** 335-343 (1995).
4. Buysse, D. J., Reynolds, C. F., Monk, T. H., Berman, S. R., & Kupfer, D. J. The pittsburgh sleep quality index: a new instrument for psychiatric practice and research. *Psychiatry Res.* **28,** 193–213 (1989).
5. Spielberger, C. D., Gorsuch, R. L., Lushene, R., Vagg, P. R. & Jacobs, G. A. *Manual for the State-Trait Anxiety Inventory.* Palo Alto, CA: (Consulting Psychologists Press, 1983).
